# Attained body mass index among children attending rural outdoor or urban conventional kindergartens

**DOI:** 10.3389/fpsyg.2023.1166512

**Published:** 2023-06-22

**Authors:** Sofus C. Larsen, Jeanett F. Rohde, Nanna J. Olsen, Jane N. Østergaard, Berit L. Heitmann, Ina O. Specht

**Affiliations:** ^1^Research Unit for Dietary Studies, Bispebjerg and Frederiksberg Hospital, The Parker Institute, Frederiksberg, Denmark; ^2^The Research Unit for General Practice and Section of General Practice, Department of Public Health, University of Copenhagen, Copenhagen, Denmark; ^3^Steno Diabetes Center Aarhus, Aarhus University Hospital, Aarhus, Denmark; ^4^The Boden Collaboration, Charles Perkins Centre, The University of Sydney, Sydney, NSW, Australia

**Keywords:** obesity, body mass index, children, kindergarten, outdoor kindergarten

## Abstract

**Objective:**

This study aimed to examine whether children in rural outdoor kindergartens had attained a lower body mass index z-score (BMIz) and were at lower risk of overweight after school entrance compared to children in urban conventional kindergartens.

**Methods:**

This is a longitudinal observational study of 1,544 children from outdoor kindergartens and 1,640 from conventional kindergartens. The mean age at kindergarten enrolment was 3.5 years (SD: 0.9) in the outdoor kindergartens and 3.6 years (SD: 1.0) in the conventional kindergartens. Anthropometry was measured after school entry by school health nurses when the children were 6 to 8 years old. Attained BMIz was included as the primary outcome. The risk of attaining overweight (including obesity) was included as a secondary outcome. Register-based information was available on potential confounding factors. Linear and logistic regression models were used to assess group differences in outcome measures.

**Results:**

Our basic models, with information on outcome, kindergarten type, and birth weight showed a borderline statistically significantly lower attained BMIz (−0.07 [95% CI: −0.14, 0.00], *P* = 0.060) and a lower risk of overweight (adjusted risk ratio: 0.83 [95% CI: 0.72, 0.97], *P* = 0.016) among children attending outdoor kindergartens. However, when adjusting for sociodemographic factors and parental BMI, there was no evidence of differences in attained BMIz (*P* = 0.153) or overweight (*P* = 0.967).

**Conclusion:**

When considering confounding factors, our findings indicate no differences in attained BMIz or risk of overweight after school entry among children attending rural outdoor kindergartens compared to those attending urban conventional kindergartens.

## Background

Between 1975 and 2016, the global prevalence of obesity among children and adolescents increased from 0.9% to 7.8% among boys and from 0.7% to 5.6% among girls (Abarca-Gómez et al., 2017). This has raised serious public health concerns as childhood obesity is associated with both short- and long-term health consequences, including negative self-evaluation, bullying, social stigma, symptoms of depression or anxiety (Puder and Munsch, 2010; Sahoo et al., 2015), numerous physical comorbidities (Llewellyn et al., 2016), and an increased risk of mortality both in early (Lindberg et al., 2020) and later adulthood (Park et al., 2012). Similar to many other health-related conditions, obesity displays a pattern of uneven distribution across populations, with socioeconomic status (SES) emerging as a key determinant (Laura et al., 2022). Once childhood obesity is established, it is difficult to reverse (Al-Khudairy et al., 2017) and children with obesity often grow up to be adults with obesity (Singh et al., 2008), strengthening the case for early intervention. However, the effect of existing prevention interventions is modest at best (Brown et al., 2019), indicating that alternative approaches may be needed to effectively promote healthy weight development in childhood.

The decline in outdoor activities among children has been suggested as a contributing factor to the growing prevalence of childhood obesity (Clements, 2004). Several cross-sectional studies have shown both outdoor play time and the quality of the outdoor environment to be inversely associated with childhood body mass index (BMI), waist circumference, and risk of obesity (Söderström et al., 2013; Ansari et al., 2015; Zhang et al., 2020; Fyfe-Johnson et al., 2021). Although there is limited evidence from longitudinal studies, findings based on a cohort of 2810 children from the Child Experiences Survey in 2006 indicate that outdoor play time was linked to decreases in children's BMI over the course of a preschool year (Ansari et al., 2015). Armstrong et al. (2015) also reported a decrease in BMI z-score (BMIz) over time among 93 overweight children involved in a family-based weight management intervention in areas with a higher density of parks or other recreational locations. Nevertheless, while some studies do indicate that outdoor activities may mitigate weight gain and prevent obesity in children, the evidence remains inconsistent as highlighted by a recent systematic review examining the relationship between nature exposure and various measures of childhood health (Fyfe-Johnson et al., 2021). Furthermore, there is a need for the development and evaluation of interventions that can be implemented on a larger scale and in a cost-effective manner.

An ideal setting for conducting childhood interventions is kindergarten, where many children spend a large part of their daily lives and may establish some of the habits and health behaviors that can track into later childhood and adulthood (Ward et al., 2015). Larger cities in Denmark and other Scandinavian countries have two different types of kindergartens: (1) the conventional urban kindergarten where the children have access to playgrounds but also spend some of their days indoor and (2) the rural outdoor kindergarten where most or all of the day is spent playing outside, often in a forest setting environment. Children attending outdoor kindergartens are often living in urban areas and thus are transported by bus from the city to the outdoor kindergarten setting (Christoffersen et al., 2014).

Combined with the unique Danish health registers and systematic measurements of height and weight performed by school health nurses, we had a unique opportunity to examine outdoor kindergartens as a potential intervention setting for preventing the development of overweight later in childhood. Our primary objective was to examine whether children who attended rural outdoor kindergartens had attained a lower BMIz and risk of overweight (including obesity) after school entrance compared to children in urban conventional kindergartens based on the first available measure of outcome recorded by school health nurses when children were aged between 6 and 8 years. As secondary objectives, we examined potential effect modification by socioeconomic status, the trajectories of attained BMIz, and the risk of overweight at 6, 7, and 8 years of age for children in outdoor kindergartens and conventional kindergartens.

## Materials and methods

### Study design

The present study was part of the “*Outdoor kindergartens—the healthier choice?”* (ODIN) project, where data were obtained from conventional kindergartens with many indoor activities and outdoor kindergartens, where most or all of the daily activities took place outside with a more pedagogic focus on nature, motor development, and outdoor and risky play (Christoffersen et al., 2014; Sederberg et al., 2023). We had information on a total of 5,077 children from the two largest municipalities in Denmark (Aarhus and Copenhagen), comprising all children who attended outdoor kindergartens (*n* = 2,434) and a subset of children who attended conventional kindergartens (*n* = 2,643). Data from Aarhus municipality were collected between 2011 and 2019, while data from Copenhagen municipality were collected between 2014 and 2019. Data from outdoor and conventional kindergartens were obtained from the same areas of parental residence. In the complete case analysis, we excluded all participants with missing data on one or more covariates (*n* = 729) or follow-up information on outcomes (*n* = 1,164), resulting in a total of 3,184 children. In total, 1,544 of these children were from outdoor kindergartens and 1,640 were from conventional kindergartens. A flowchart illustrating the inclusion/exclusion of individuals can be found in the supporting information (Supplementary Figure S1).

### Ethical considerations

Permission from the two municipalities to send information to Statistics Denmark was granted. Ethical permission was decided not to be relevant by the Ethical Committee of the Capital Region of Denmark (journal no.: H-19053587). Permission from the Capital Region Data Agency and the Danish Patient Safety Authority was granted (journal nos.: P-2020-54 and 31-1521-8, respectively).

### Exposure

In the present study, we included the kindergarten type as the exposure (outdoor/conventional). In Danish conventional kindergartens, children spend time both indoors (playing with toys, drawing, etc.) and outdoors in the kindergarten playground. Before lunch, the activities are structured by the kindergarten teachers and could be both inside and outside. After lunch, the children are most often sent outside (for ~2 h) to play in the playground. The children decide for themselves what they want to do; however, the kindergarten teachers also organize activities in the playground, which the children can choose to participate in.

The outdoor kindergartens are in an environment surrounded by nature, and children spend almost full time outdoors in all seasons. Every morning parents dropped off the children at a collection point, typically the address of the conventional kindergarten setting, and from there, the children were driven by bus for 30–60 min to the outdoor kindergarten setting. Outdoor kindergartens and conventional kindergartens differ in their environmental setting with a larger outdoor area often in forests or other natural environments among outdoor kindergartens than conventional kindergartens. The children and kindergarten teachers spend most of the day outside in the outdoor kindergarten, and nature and motor development are often pedagogic areas of interest (Rohde et al., 2023).

### Outcomes

Almost all Danish schoolchildren undergo a health examination upon entering primary school, and to a lesser extent, during the subsequent early years of primary school. These data were recorded and uploaded by school health nurses. Height was measured in whole centimeters using a stadiometer. Weight was measured in kilograms with one decimal place. Approved scales were labeled as OIML class III. Measurements were taken without shoes and in light clothing. From this, we objectively measured information on height and weight when the children were 6 to 8 years old. The collected data were checked for plausibility. BMI was calculated as weight in kilograms per height in meters squared. We then generated BMIz using a power transformation in increments of 0.1 years, applying national reference scores to the study population. The first available measure of attained BMIz was included as our primary outcome, while the first available measure of attained overweight (including obesity) (defined as a BMIz>1 [yes/no]) was included as a secondary outcome.

### Covariates

Gender of the child (boys/girls), birth weight (g), preterm birth (yes/no), maternal age (years), maternal pre-pregnancy BMI (kg/m^2^), and information on maternal smoking during pregnancy (yes/no) were obtained from the *Danish Medical Birth Register*. Information on maternal education was obtained from *Statistics Denmark* and included in the present study as *basic* (basic school 8th−10th class), *short* (general upper–secondary education, short-cycle higher education, or vocational education and training), *medium* (medium-cycle higher education or bachelor), and *long* (long-cycle education and PhD). Information on maternal country of origin (Western/non-Western) was also obtained from *Statistics Denmark*. Age at kindergarten enrolment (years) and total time spent in kindergarten (years) were obtained from the municipalities.

#### Additional covariates only included in sensitivity analyses

Information on paternal education and country of origin was obtained from *Statistics Denmark*, and the same categories were included for maternal education and country of origin. In addition to birth weight, a subset of 2,681 children had weight and length measured during the first year of life by health nurses at the home of the child. From this information, we included the last available measure of BMIz prior to kindergarten enrolment.

### Statistical analysis

A detailed statistical analysis plan, including estimates of power and precision, was circulated to all authors prior to conducting any statistical analyses (Supplementary material S1).

Linear regression models were constructed to examine the mean difference in BMIz between children in outdoor kindergartens and conventional kindergartens based on the first recorded measure after school entry. We constructed a basic model with information on BMIz, kindergarten type, and birth weight, and a fully adjusted model with added information on maternal age, maternal education, maternal country of origin, maternal smoking during pregnancy, maternal pre-pregnancy BMI, preterm birth, child's age at kindergarten enrolment, and total number of years spent in kindergarten.

Using the same adjustment strategy, we constructed logistic regression models to examine the risk of attaining overweight among children in outdoor kindergartens compared to conventional kindergartens based on the first recorded measure after school entry. We used the STATA postestimation command *adjrr* to attain adjusted estimates of absolute risks in addition to adjusted risk differences (aRD) and adjusted risk ratios (aRR) with corresponding 95% confidence intervals (95% CI) (Norton et al., 2013).

#### Between-group differences in subgroups with low or high levels of maternal education

Low SES is a strong predictor of childhood obesity, and previous results from the ODIN project have shown parental education to be higher for children attending outdoor kindergartens (Specht et al., 2022). Thus, in addition to adjustment for maternal education, we conducted subgroup analyses specifically for children of mothers with a low level of education (defined as mothers with a basic or short education) and a high level of education (defined as mothers with a medium or long education). Effect modification by the maternal education level was tested by adding the new 2-category education variable and a product term (kindergarten type × education) to the fully adjusted models after removing the original 4-category education variable.

#### Trajectories of attained BMIz and overweight

The same overall strategy was used to explore trajectories of attained BMIz and overweight at specific ages after school entry (6, 7, and 8 years of age). These analyses were restricted to children with at least one measurement of BMIz at 6 years of age. A last observation carried forward approach was then used for children with missing information at 7 and/or 8 years of age.

#### Sensitivity analyses

Since BMIz considers differential BMI trajectories by sex and age, we did not include sex as a potential confounder. However, the effect modification of sex was tested by adding the variable *sex* and a product term (kindergarten type × sex) to the fully adjusted models.

We had no baseline measure of outcome available, but in addition to birth weight, most of the children had weight and length measured during the first year of life. From this, we added the last available measure of BMIz prior to kindergarten enrolment as a covariate.

We used maternal education as a proxy for the child's SES. Similarly, we used maternal country of origin as a proxy for the geographic origin of the child. However, analyses were also conducted adding paternal information to the models.

Our primary analyses were conducted on the complete case sample of 3,184 individuals. However, a sensitivity analysis using multiple imputations by chained equations (MICE) was implemented on the full sample of 5,077 individuals.

Finally, we examined a potential dose–response relationship between time spent in kindergarten (continuous variable) and attained BMIz and tested for effect modification of kindergarten type by adding a product term to the model (years in kindergarten × kindergarten type).

All statistical tests were two-sided with a significance level of 0.05. All statistical analyses were performed using Stata/SE 16 (StataCorp LP, College Station, Texas, USA). Figures were produced using SigmaPlot 13.0 (San Jose, CA, USA).

## Results

Characteristics of the included children can be found in Table 1. A total of 1,544 children from outdoor kindergartens and 1,640 from conventional kindergartens were included in the primary analysis.

**Table 1:** Participant characteristics of children in outdoor kindergartens vs. conventional kindergartens.

	**Outdoor kindergarten (*n* = 1,544)**	**Conventional kindergarten (*n* = 1,640)**
	**Mean (SD)**	**Mean (SD)**
Girls, *n* [%]	678 (43.9)	807 (49.2)
Age at kindergarten enrolment, years	3.5 (0.9)	3.6 (1.0)
Age at first school measurement, years	6.5 (0.5)	6.5 (0.5)
Time spent in kindergarten, years	2.4 (1.0)	2.3 (1.0)
Birth weight, g	3,519 (515)	3,449 (546)
Born preterm, *n* [%]	70 (4.5)	116 (7.1)
Maternal age at pregnancy, years	32.1 (4.6)	31.4 (4.9)
Maternal pre-pregnancy body mass index, kg/m^2^	22.4 (3.4)	23.1 (4.1)
**Maternal education**		
Basic, *n* [%]	84 (5.4)	216 (13.2)
Short, *n* [%]	280 (18.1)	429 (26.2)
Medium, *n* [%]	466 (30.2)	441 (26.9)
Long, *n* [%]	714 (46.2)	554 (33.8)
**Maternal country of origin**		
Non-Western, *n* (%)	56 (3.6)	324 (19.8)
Mother smoked during pregnancy, *n* (%)	103 (6.7)	155 (9.5)

Results are presented as mean (SD) unless otherwise stated.

The basic models with information on outcome, kindergarten type, and birth weight did not yield any statistically significant difference in BMIz at school entry (−0.07 [95% CI: −0.14, 0.00], *P* = 0.060) between the two types of kindergartens. However, there was a statistically significant lower risk of overweight (aRR: 0.83 [95% CI: 0.72, 0.97], *P* = 0.016) among children in outdoor kindergartens compared to conventional kindergartens. In the full model, with added information on sociodemographic factors, we found no differences in BMIz (0.06 [95% CI: −0.02, 0.12], *P* = 0.153) or risk of overweight (aRR: 1.00 [95% CI: 0.86, 1.17], *P* = 0.967) between kindergarten types (Table 2).

**Table 2:** Attained BMI z-scores and risk of overweight among children in outdoor kindergartens vs. conventional kindergartens.

	**Mean or % of participants (SE)**		**Estimated difference**	
	**Outdoor kindergarten**	**Conventional kindergarten**	**Mean difference or aRD and aRR (95% CI)**	***P*** **-value**
**BMIz**				
Basic model^a^	0.05 (0.03)	0.12 (0.03)	−0.07 (−0.14, 0.00)	0.060
Full model^b^	0.11 (0.03)	0.06 (0.02)	0.06 (−0.02, 0.12)	0.153
**Overweight**				
Basic model	16.47% (0.93)	19.77% (0.98)	aRD: −3.29% (−5.96, 0.63) aRR: 0.83 (0.72, 0.97)	0.016 0.016
Full model	18.18% (0.99)	18.13 (0.92)	aRD: 0.06% (−2.66, 2.77) aRR: 1.00 (0.86, 1.17)	0.967 0.967

aRD, adjusted risk difference; aRR, adjusted risk ratio; BMIz, body mass index Z-score.

^a^Model with information on outcomes, kindergarten type, and birth weight.

^b^Added information maternal age, maternal education, maternal country of origin, maternal smoking during pregnancy, maternal pre-pregnancy body mass index, born preterm, age at kindergarten enrolment, and total number of years spent in kindergarten.

**Figure 1:**
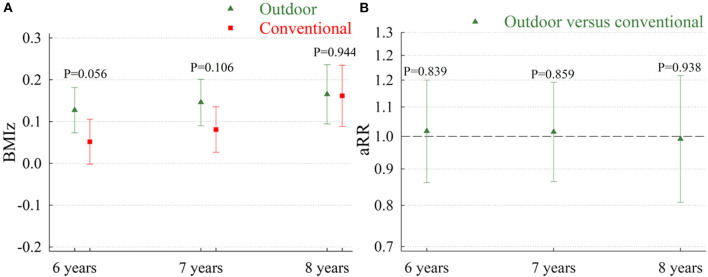
Attained BMI z-scores **(A)** and adjusted risk ratios for overweight **(B)** among children in outdoor kindergartens vs. conventional kindergartens. Results stratified by maternal education. aRR, adjusted risk ratios; BMIz, body mass index Z-score; SES, socioeconomic status. Low maternal education is defined as mothers with a basic or low education. High education is defined as mothers with a medium or high education. Results from the model with information on outcome, kindergarten type, birth weight, maternal age, maternal country of origin, maternal smoking during pregnancy, maternal pre-pregnancy body mass index, preterm birth, age at kindergarten enrolment, and total number of years spent in kindergarten. Test statistics for BMIz: *P*-value for effect modification by education = 0.512. Test statistics for aRR: *P*-value for effect modification by education = 0.154.

**Figure 2:**
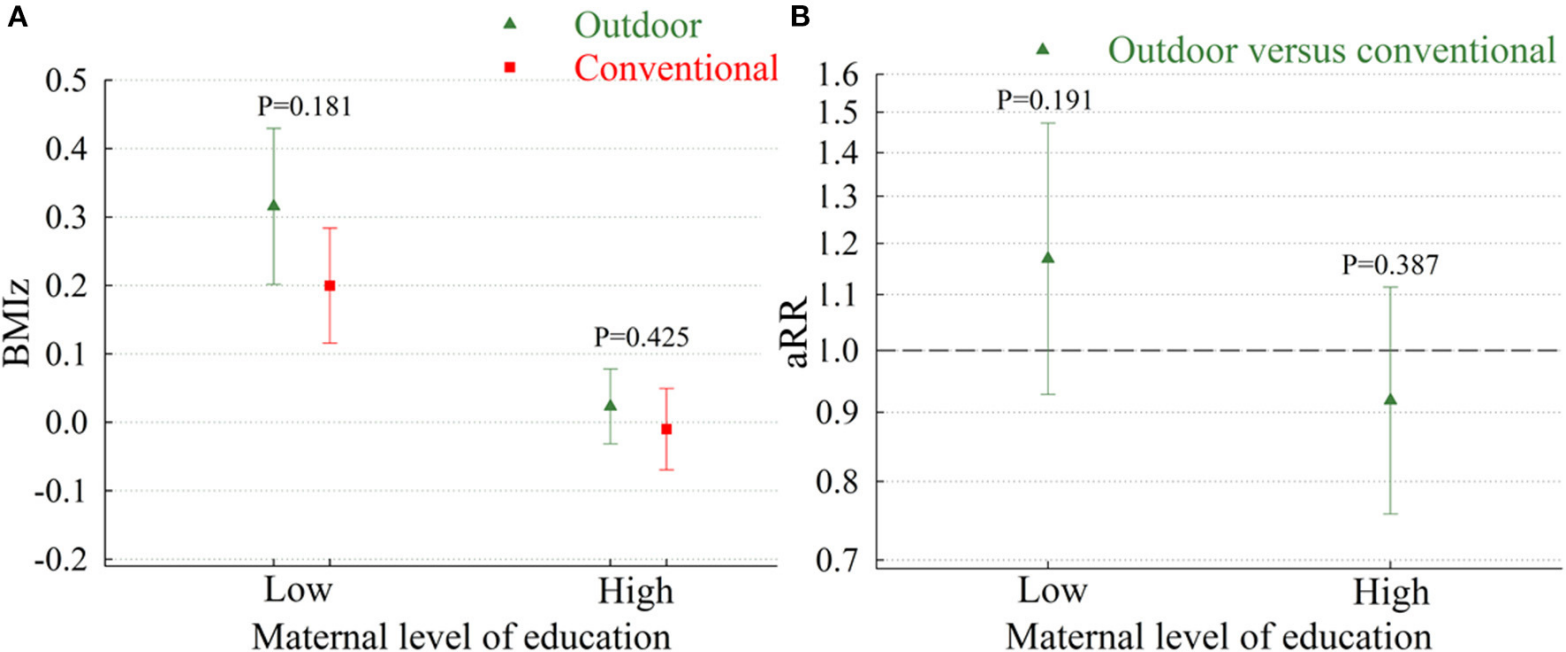
Trajectories of attained BMI z-scores **(A)** or overweight **(B)** at 6, 7, and 8 years of age among children in outdoor kindergartens vs. conventional kindergartens. BMIz, body mass index Z-score; aRR, adjusted risk ratios. Results from the model with information on outcomes, kindergarten type, birth weight, maternal age, maternal country of origin, maternal smoking during pregnancy, maternal pre-pregnancy body mass index, preterm birth, age at kindergarten enrolment, and total number of years spent in kindergarten.

For both types of kindergartens, the highest attained BMIz after school entry was observed among children of mothers with a low level of education, but we found no effect modification by maternal education in relation to the association between kindergarten type and attained BMIz (*P* = 0.512) or risk of overweight (*P* = 0.154). In support of this, the fully adjusted models showed no between-group differences in BMIz or risk of overweight in subgroups with mothers of low or high educational level (Figure 1). Similarly, when comparing trajectories of attained BMI z-scores or overweight at 6, 7, and 8 years among children in outdoor kindergartens and conventional kindergartens, we did not find any statistically significant between-group differences (Figure 2).

### Sensitivity analyses

We found no effect modification by sex in relation to BMIz (*P* = 0.486) or aRR of obesity (*P* = 0.800). After adding the last available measure of BMIz prior to kindergarten enrolment (Supplementary Table S1) or paternal information on education and country of origin (Supplementary Table S2) as covariates, our results remained statistically non-significant. Similarly, analyses where missing information was handled through multiple imputations also remained statistically non-significant (Supplementary Table S3). Finally, we found no association between years spent in outdoor or conventional kindergartens and attained BMIz and no evidence of effect modification by kindergarten type (*P* = 0.571) (Supplementary Figure S2).

## Discussion

In our study, we investigated whether attending rural outdoor kindergartens resulted in a lower attained BMIz and risk of overweight after school entry compared to urban conventional kindergartens. In a basic model with information on outcome, kindergarten type, and birth weight, we found a lower risk of overweight after school entry among children in rural outdoor kindergartens compared to urban conventional kindergartens. However, when adding sociodemographic factors to the models, we found no differences in attained BMIz or risk of overweight between the two types of kindergartens, suggesting some degree of confounding in the basic model. We found no evidence that differences were modified by maternal education.

Our earlier research has indicated that children exhibit higher levels of physical activity during their attendance in outdoor kindergartens in comparison to conventional ones. We also observed compensatory periods of inactivity outside of kindergarten hours, which suggests that the overall effect on activity levels may be limited. However, it is important to note that in this study, the SENS motion^®^ device was utilized, which had not yet undergone validation specifically in children (Rohde et al., 2023). While the present study cannot confirm or refute causal effects, our results also support that the potential effects of outdoor kindergartens on children's activity levels or other potential mediators of body weight regulation are insufficient to prevent overweight or obesity at school entry.

Similar to Denmark, outdoor kindergartens are also part of the Swedish daycare system. Based on cross-sectional data, Söderström et al. (2013) have previously found that Swedish children attending kindergartens with a high-quality outdoor environment less frequently had overweight than those attending kindergartens with a low-quality outdoor environment. However, due to the cross-sectional design and the lack of adjustment for potentially important covariates, including parental BMI and socioeconomic status, the causal nature of this finding is highly uncertain. We found no previous studies directly comparing measures of attained adiposity in school-aged children who had attended outdoor kindergartens and conventional kindergartens. However, as pointed out in a recent systematic review on nature and child health by Fyfe-Johnson et al. (2021), only one-third of the published studies (*n* = 45) found a lower risk of overweight among children with higher levels of nature exposure. In our study, we found that children attending outdoor kindergartens had a lower risk of attaining obesity at school entry than children in conventional kindergartens before adjustment for important covariates such as familial BMI and SES. Indeed, the adjustment completely attenuated the observed associations, suggesting confounding from these factors. This further suggests that insufficient adjustment for confounding may have biased many of the positive results seen in previously published studies (Fyfe-Johnson et al., 2021).

We also examined effect modification by maternal education as low SES is a strong predictor of childhood obesity, and previous results from the ODIN project have shown parental education to be higher among children attending outdoor kindergartens (Specht et al., 2022). Although not specifically focused on obesity, there is also some evidence suggesting that children in low socioeconomic areas may have the greatest psychological benefits from exposure to high-quality outdoor environments (Wells and Evans, 2003). We found no evidence of effect modification in our analysis, but this could potentially also be due to relatively high socioeconomic equality in the Danish population.

Our study has several strengths, including a prospective design with objectively measured information on study outcomes. The Danish Health Data Authority is responsible for the national health registers and for maintaining and developing standards and classifications, as a result, the standardization and digitization data from the Danish health registers are known to be of very high quality (Thygesen et al., 2011). We also had a relatively large sample size, providing us with sufficient statistical power to detect even a small between-group difference in BMIz and risk of overweight. In addition, we had information on several covariates from the unique Danish health registers, including child and parental sociodemographic factors, allowing us to adjust for potential confounders.

On the other hand, our study also has some limitations. We had no information available on BMIz at kindergarten enrolment, which increases the risk of reverse causality. However, since we found no evidence of between-group differences in the adjusted models, including the sensitivity analysis with added information on the last available measure of BMIz prior to kindergarten enrolment, it seems unlikely that adding a baseline measure of outcomes to the model would have had a substantial impact on our results. We have previously shown a high degree of selection related to parental choice of kindergarten (Specht et al., 2022), and thus, we can also not exclude that some unmeasured or residual confounding has remained. As an example of this, we had no information available on the daily routines of the included children/families. We also had no information available on body composition, and we can therefore not rule out a relevant between-group difference in attained fat or lean mass. Since we relied on data available from the Danish health registers, we used BMIz as a proxy for adiposity. It is important to note that this measure is not without limitations as previous studies among children have suggested only a moderate correlation between BMIz and other measures of excess body fat, including skinfold thickness measurements and dual-energy X-ray absorptiometry (DXA) (Freedman et al., 2017; Monasor-Ortolá et al., 2021).

Finally, our results originate from children attending kindergartens in two large Danish municipalities and may not be generalizable to children from other areas, and we had a relatively large proportion of missing data. However, we found almost identical results when using multiple imputations to deal with missing data. Moreover, as we found no evidence of effect modification by maternal education or sex of the child, it seems unlikely that results would be vastly different in Danish populations with other sociodemographic distributions.

In conclusion, when considering confounding factors, our findings indicate no differences in attained BMIz or risk of overweight after school entry among children attending rural outdoor kindergartens compared to those attending urban conventional kindergartens. It is important to note that our study was observational in nature, and therefore, caution is advised when interpreting these results. Before considering any changes to current kindergarten practices, we strongly recommend conducting randomized trials to confirm the validity of our findings.

## Data availability statement

The original contributions presented in the study are included in the article/Supplementary material, further inquiries can be directed to the corresponding author.

## Ethics statement

The studies involving human participants were reviewed and approved by the Ethical Committee of the Capital Region of Denmark (journal no.: H-19053587). Permission from the Capital Region Data Agency and the Danish Patient Safety Authority was granted (journal nos.: P-2020-54 and 31-1521-8, respectively). Written informed consent from the participants' legal guardian/next of kin was not required to participate in this study in accordance with the national legislation and the institutional requirements.

## Author contributions

SL conceptualized study, created dataset, carried out statistical analyses, and drafted the initial statistical analysis plan and manuscript. NO and JØ critically reviewed and revised statistical analysis plan and manuscript. BH and IS conceptualized study, acquired funding for the project, and critically reviewed and revised statistical analysis plan and manuscript. All authors contributed to the article and approved the submitted version.
